# Electrophysiologic evaluation of the visual pathway at different depths of sevoflurane anesthesia in diabetic rats

**Published:** 2018

**Authors:** Daniela Adriana Iliescu, Alexandra Ciubotaru, Mihai Aurelian Ghiţă, Adrian Marius Păun, Tudor Ion, Leon Zăgrean

**Affiliations:** * Physiology Department, “Carol Davila” University of Medicine and Pharmacy, Bucharest, Romania; ** Ophthalmology Department, “Dr. Carol Davila” Central Military University Emergency Hospital, Bucharest, Romania; *** Ophthalmology Department, University Emergency Hospital, Bucharest, Romania

**Keywords:** visual evoked potential, electroretinography, diabetes, sevoflurane

## Abstract

Our study investigated the changes produced by diabetes on the visual pathway in a Wistar rat model. The impact of diabetes at 10 weeks after intraperitoneal streptozotocin (STZ) injection was evaluated through electrophysiological methods like visual evoked potentials (VEP) and electroretinogram (ERG). VEP and ERG were recorded simultaneously under different sevoflurane anesthetic depths. In all tested concentrations, sevoflurane affected the amplitude and latency of VEP and ERG component elements. With increasing anesthetic depths, sevoflurane increased the latencies of VEP N1, P1 and N2 peaks and ERG a- and b- waves in both control and diabetic animals. On the other hand, the amplitude of VEP showed enhancement in higher concentrations of sevoflurane, contrariwise to the drop of amplitude seen in the ERG. Diabetes additionally increased the latencies of VEP peaks and decreased the N1-P1 amplitude of the VEP when compared to control at the same anesthetic depth. The a- and b- waves were also delayed by diabetes at 10 weeks post-STZ diabetic induction, with the exception of highly profound anesthetic depth in which the result for the b wave were conflicting. We found a reduction in amplitude of the a-b wave in diabetic animals, when ERG was recorded under 6% and 8% sevoflurane concentration. In conclusion, neurophysiological studies like VEP and ERG are useful in the assessment of retinal and optic nerve dysfunctions produced by diabetes, yet considering the alterations that occur during anesthesia if this is used.

## Introduction

The purpose of our study was to investigate the influences of moderate and deep levels of sevoflurane anesthesia on optic nerve conduction and retinal function in diabetic rats. Our research followed the changes of VEP (visual evoked potential) and ERG (electroretinogram) recorded under sevoflurane anesthesia in diabetic animals. More than 60% of the diabetic patients were expected to develop a form of retinopathy in the first decade of the disease. This could be the cause of several abnormalities: increased polyol pathway, activation of protein kinase C pathway, increased expression of growth factors, hemodynamic changes, accelerated formation of advanced glycation end products, oxidative stress, or activation of the renin-angiotensin-aldosterone system [**1**]. The effects of these abnormalities could lead to retinal microaneurysms with microhemorrhages or microthrombosis, hyaline deposits or angiogenesis. Recent studies have pointed towards the role of activated microglia in diabetic patients in the release of pro-inflammatory mediators and proliferation resulting in severely affected retinal neurons [**2**]. Besides diabetic retinopathy, optic nerve neuropathy manifested as a delay in neural conduction of the post-retinal visual pathways, another complication of diabetes that leads to impaired vision. 

Neurophysiological techniques are available to assess both optic nerve and retinal dysfunction generated by diabetes [**3**]. VEP is an electroencephalographic pattern, which shows the electrical signals generated by the occipital cortex, during flash light stimulation of the retina. The amplitude of the signal is related to the integrity of the visual pathways and of the occipital area itself. VEP abnormalities have been described in patients with Diabetes Mellitus, more important in those who already suffer from diabetic retinopathy [**4**]. On the other hand, ERG is a noninvasive method that assesses the bioelectrical response to the visual stimuli of the retina, reflecting events associated to photoreceptors, bipolar cells, amacrine cells and Muller cells [**3**]. Changes produced in a and b wave amplitude and latency can have a role in scaling the severity and prognosis of the diabetic disease [**5**]. The clinical importance of our study is that exploration of VEP and ERG during anesthesia can be disturbed by the depth of the anesthesia, especially in diabetic patients.

## Materials and methods 

For our study, we used 16 male Wistar rats of approximately 3 months old (medium weight 350 g), which were reared in a normal 12h light/ dark cycle, with food and water ad libitum. Rats were divided equally and randomly into two groups, control (non-diabetic), and diabetic. For each group we used 2%, 4%, 6% and 8% inhalational concentration of sevoflurane for anesthesia and recorded simultaneous ERG and VEP, during flash stimulation.

***Electrodes settings***

Both ERG and VEP recordings were performed using electrode tips from nickel-chromium alloy (ni80cr20, diameter 0.15 mm). The electrodes for VEP recordings were implanted through and fixed in the cranial bones, one week before the rats in diabetic group were made diabetic, in order to give time to the surgical wound to heal without complications. The surgical procedure was performed under anesthesia with intraperitoneal solution of chloral hydrate (0.4g/ Kgc). The withdrawal reflex to noxious stimuli was periodically assessed during the procedure, and we adjusted the chloral hydrate dose whenever required. The actual surgery had the following steps: a median incision was performed on the scalp, then the tegument and connective tissue were sideways shifted, periosteum was easily scraped from the bone, the head of the rat was fixed in a stereotaxic apparatus, the bregma was identified, two trepanations were done for each electrode using a dental drill, retaining between them 1 mm bone bridge for anchoring the electrode, the electrodes were placed in their final position crossing the epidural space from one trepanation to the immediately close one and the last step was the suturing the scalp around the electrodes. The sites of the electrodes were decided in agreement with the atlas of Paxinos and Watson (Paxinos G, 1998). Two active electrodes were placed over the right and left occipital area (6 mm dorsal to bregma, 4 mm lateral to midline). The reference electrode was placed over the olfactory bulb (7 mm anterior to bregma, on the midline).

For the ERG, the electrodes were positioned as it follows the active electrode on the stimulated eye, the reference on the mouth and the ground on the tail. The active and reference electrodes were loop-shaped nickel-chromium wires. The active electrode was placed around the rim of the eyeball in the conjunctival sack of the stimulated eye and the reference electrode in the mouth of the animal. Before the ERG recording we instilled oxibuprocaine hydrochloride (Benoxi 4 mg/ ml, UnimedPharma), a corneal anesthetic, and tropicamide (Mydriacyl, 0.5%, Alcon). 

***Diabetic induction***

We induced diabetes in the diabetic group by specifically destroying the pancreatic β-cells, achieved by an intraperitoneal injection of streptozotocin (STZ) at a dose of 50 mg/ kg body weight, after overnight fasting. Two days after the injection, the glycemia was measured with a glucometer (ACCU-CHEK Performa Nano; Roche, Germany). Rats were considered diabetic only if glycemic values were over 300 mg/ dl. We kept monitoring glycemic values at every two weeks. 

***ERG and VEP recordings***

All our recording settings were placed in a dark and soundproof room. During anesthesia induction, the rat was placed for 1 minute in a custom-built anesthetic induction chamber where 8% sevoflurane was vaporized in 2L/ min oxygen. Afterwards, the animal was immediately placed in a gas delivery system. ERG and VEP electrodes were set in the above-mentioned positions, connected to Biopac MP150 system. We simultaneously recorded ERG and VEP for every rat, at each inhalational concentration of sevoflurane (2%, 4%, 6% and 8%), consecutively. After switching from one concentration to another, a waiting period of 5 minutes was followed so that the anesthetic concentration in the rat system would equalize the one delivered. In the diabetic group, ERG and VEP were recorded at 10 weeks after streptozotocin induced diabetes.

Light flashes were delivered at a rate of 30 stimuli/ min with o a duration of 0.015 s, by a LED placed at 1 cm distance from the stimulated eye. Both retinal and visual cortex responses to the stimuli were recorded in 300 s epochs. The signals were band-pass filtered between 1 and 100 Hz, amplified by 10000 times. Data was acquired and digitalized at 1000fps on a computer through AcqKnowledge 4.2 software. Amplitudes and latencies of the ERG a- and b-waves and VEP N1, P1, N2 peaks were analyzed using IBM SPSS Statistics 22. Mean values and standard deviation were calculated. One-way ANOVA and unpaired T-test were used to compare the differences between groups (p<0.05 was interpreted as statistically significant).

**Fig. 1:**
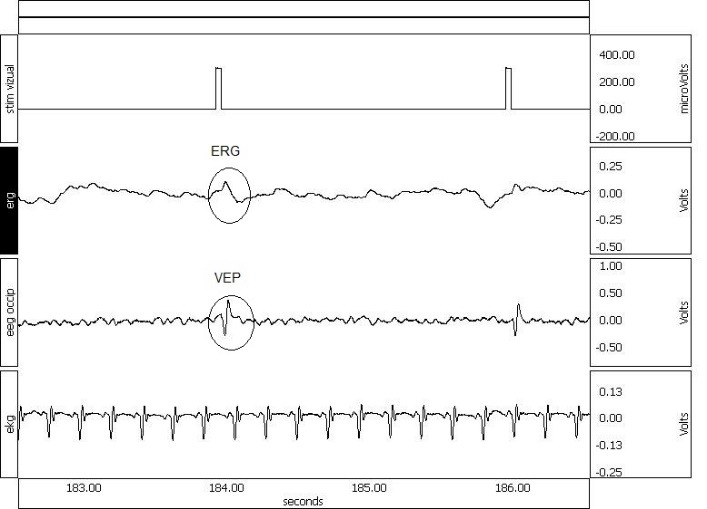
Spontaneous activity of ERG, occipital EEG, and EKG recorded under 4% sevoflurane during flash stimulation. Please note that ERG and VEP can be identified after light pulses without averaging. The EEG shows burst-suppression pattern that facilitated VEP appearance

**Fig. 2:**
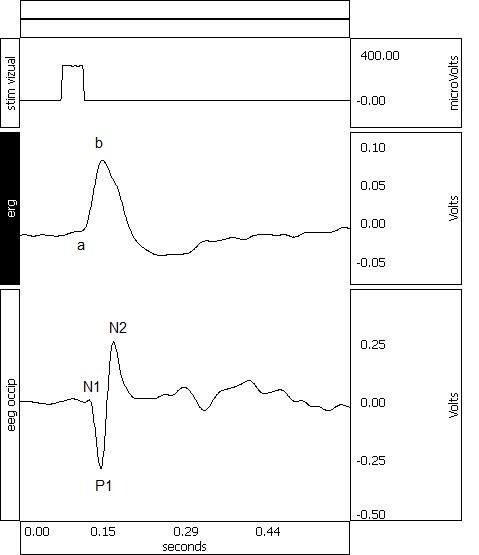
Representative ERG and VEP obtained by averaging of a 300 sec sample under 4% sevoflurane; a and b waves could be identified for ERG and N1, P1 and N2 peaks for VEP

## Results

The glycemic values in the diabetic group were maintained over the accepted threshold (>300 mg/ dl) during the whole experiment. Animals that did not achieve > 300 mg/ dl after 3 days post-STZ injection were excluded from the study. Diabetic rats developed polyuria and polydipsia and decreased in weight over the course of the 10-week experiment.

**Table 1:** Glycemic values obtained in NDM and DM groups during the experiment (NDM-non-diabetic, DM–diabetic)

Glycemia (mg/ dl)*	Baseline	2 weeks**	4 weeks**	6 weeks**	8 weeks**	10 weeks **
NDM Group	122±8	116±5	118±10	117±8	121±13	122±8
DM Group	510±66	542±12	499±62	489±28	495±37	481±31
*Mean value ± standard deviation						
** Period after STZ injection						

Sevoflurane affected the amplitude and latency of VEP and ERG component elements in all tested concentrations. Sevoflurane increased the latencies of N1, P1 and N2 VEP peaks (statistically significant for both groups, p<0.05) with increasing anesthetic depths. When diabetic and control groups were compared, N1 and N2 latency values showed a statistically significant increase in diabetic animals for all analyzed sevoflurane concentrations, except for 6% (p>0.05). For P1 peak, diabetes increased the latency in all four studied anesthetic concentrations. 

On the other hand, the amplitude of N1-P1 and P1-N2 of the VEP showed enhancement with higher concentrations of sevoflurane. The increase of amplitude of the VEP recorded with deeper anesthesia levels reached statistical significance only for N1-P1, both for non-diabetic and diabetic groups. Even though the same tendency for amplitude enhancement with higher anesthetic concentrations was observed in diabetic animals, when compared to the non-diabetic group, the amplitude of N1-P1 was decreased at the same level of anesthesia (p<0.05). For the P1-N2 amplitude, this decrease was observed only for the 8% sevoflurane level.

**Fig. 3:**
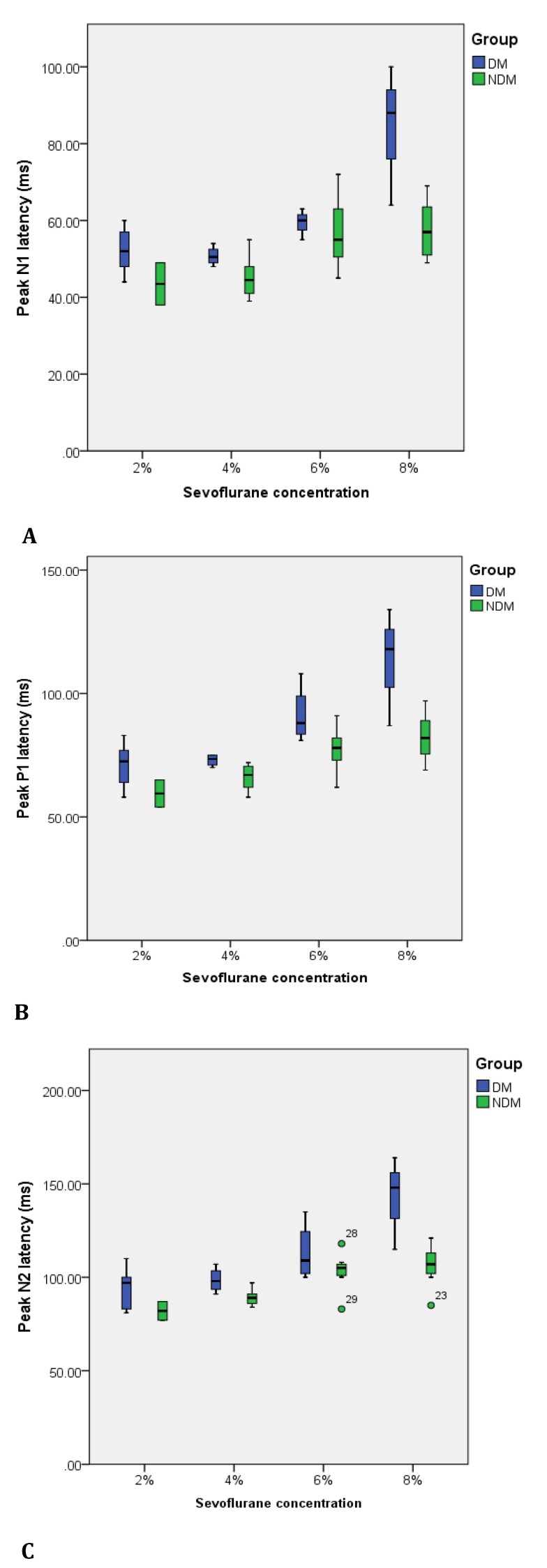
Variability of N1 (A), P1 (B) and N2 (C) peak latencies of the VEP in relation to sevoflurane anesthetic concentration for NDM (non-diabetic) and DM (diabetic) groups

**Table 2:** Changes of VEP amplitude (N1-P1 and P1-N2 amplitude) for NDM (non-diabetic) and DM (diabetic) groups in accordance with sevoflurane anesthetic concentration

NDM Group			
Sevoflurane concentration		N1-P1 Amplitude (μV)	P1-N2 Amplitude (μV)
2%	Mean	148.6667	252.3333
	Std. Deviation	72.55251	108.05122
4%	Mean	186.7500	230.0000
	Std. Deviation	139.30392	186.03046
6%	Mean	330.7500	281.0000
	Std. Deviation	237.26409	267.14540
8%	Mean	705.6667	1006.3333
	Std. Deviation	514.86050	603.99365
DM Group			
Sevoflurane concentration		N1-P1 Amplitude (μV)	P1-N2 Amplitude (μV)
2%	Mean	116.5000	311.5000
	Std. Deviation	65.76093	246.78027
4%	Mean	168.8750	359.6250
	Std. Deviation	104.66469	212.72312
6%	Mean	229.9091	345.2727
	Std. Deviation	265.82906	427.88388
8%	Mean	387.4286	537.1429
	Std. Deviation	344.43957	539.82480

The latencies of a and b ERG waves increased with anesthetic deepening for both non-diabetic and diabetic groups (except for a wave latency in the control group, where no statistical difference was noted between recordings under 6% and 8% sevoflurane concentrations). When the two groups were compared at various anesthetic depths, the diabetic group showed increased a wave latency (statistical significant for 2%, 4% and 8% sevoflurane concentrations, p<0.05) and b wave latency (statistical significant for 2% and 4% sevoflurane concentrations, p<0.05). On the other hand, the a-b wave amplitude decreased with increasing anesthetic depth. Diabetes diminished even more the amplitude for 6% and 8% sevoflurane concentrations.

**Fig. 4:**
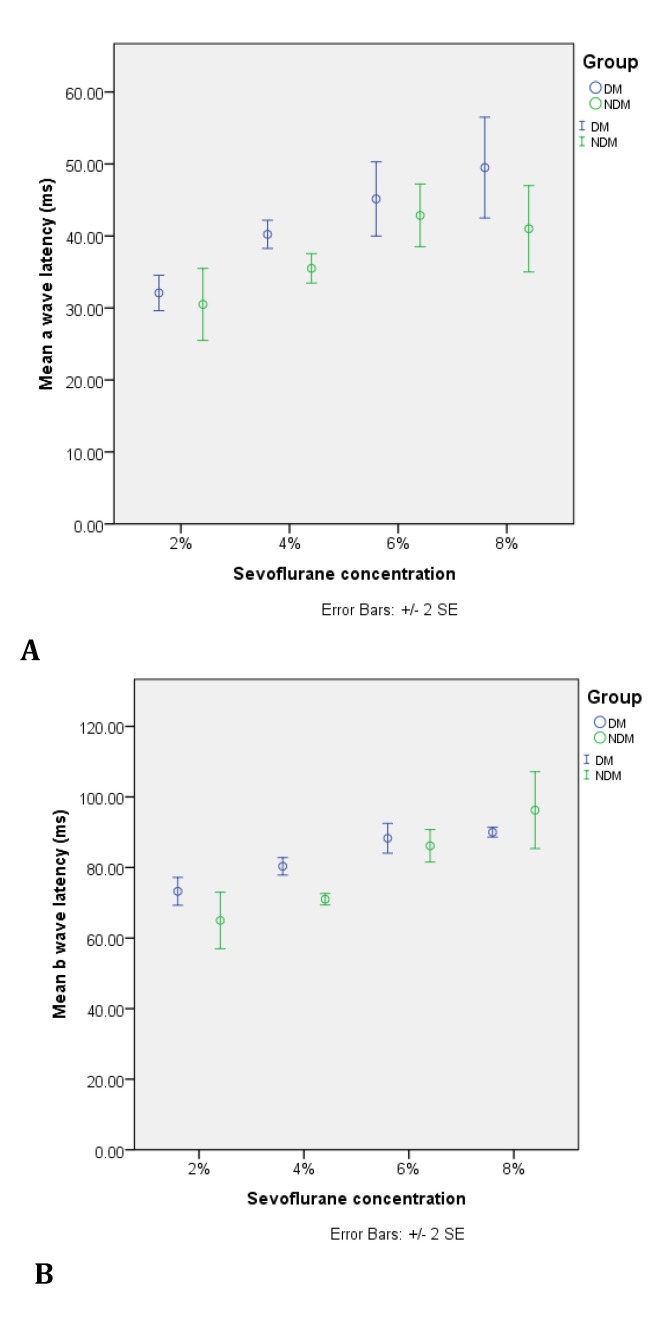
Variability of a wave (A), and b wave (B) latencies of the ERG in relation to sevoflurane anesthetic concentration for NDM (non-diabetic) and DM (diabetic) groups

**Fig. 5:**
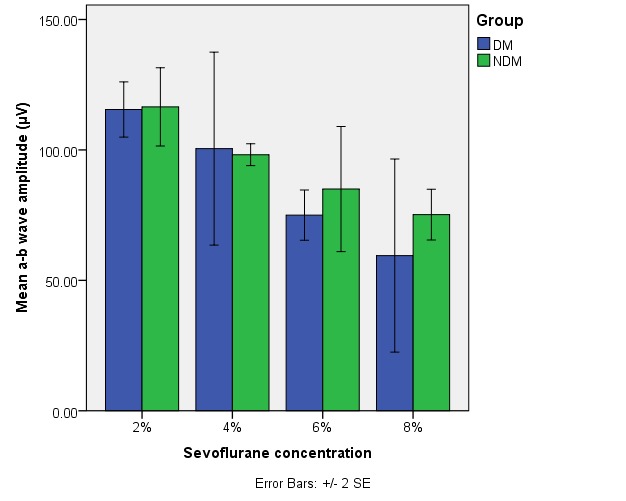
Changes of a-b wave amplitude of the ERG for NDM (non-diabetic) and DM (diabetic) groups in accordance with sevoflurane anesthetic concentration

## Discussions 

Our results supported the fact that sevoflurane and diabetes affect the VEP and ERG during medium and deep anesthetic levels. High doses of sevoflurane increase the N1, P1, and N2 peak latencies of the VEP but also potentiate the amplitude of the VEP in a dose-dependent manner in both control and diabetic groups, especially for the N1-P1 amplitude. The P1-N2 amplitude showed a great increase when the highest sevoflurane concentration was reached (8% inhalational concentration) but the amplitude difference between 2%, 4%, and 6% was not prominent. Overall, higher concentrations of sevoflurane increase latency and enhance amplitude of VEP. VEP changes, produced by volatile anesthetics, including sevoflurane, that show increased latency with more profound anesthesia, have been reported in literature, and are well-known facts [**6**-**9**]. On the other hand, our results showed an unexpected enhancement of VEP amplitude with increasing concentration of sevoflurane, suggesting a facilitated visual cortical reactivity to light stimulation during increased anesthetic depth. As most volatile anesthetics do, sevoflurane silences the brain cortical activity but as indicated by our results it also facilitates the sensory evoked responsiveness of the occipital cortex to light stimulation. This finding is similar to recent research results that confirm the increment of visual sensory response during cortical silencing produced by increased sevoflurane anesthesia. One possible suggested explanation is that in awake state, the high spontaneous activity of the brain processed in the occipital cortex depresses the input and output synapses, inhibiting the generation of the VEP. On the contrary, anesthetics like sevoflurane would potentiate the response to visual stimulation by counteracting the synaptic depression [**10**]. Older reports are not in agreement with these results, showing a decrease of VEP amplitude with an increase in sevoflurane anesthetic concentration [**7**,**8**].

It has been shown that diabetes is an additional stress factor for the optic nerve function causing optic neuropathy at 10 weeks after STZ injection. This has been proven by increased latencies of the N1, P1, and N2 peaks and by decreased N1-P1 amplitude of the VEP when compared to control at the same anesthetic depth. For the P1-N2 amplitude, we found an actual increase for diabetic animals at 2%, 4%, and 6% sevoflurane concentrations but a significant decrease at 8%. At increasing anesthetic depths in the diabetic group, we found the same tendency of amplitude enhancement of VEP as in the control group. Thus, we conclude that in the diabetic group, we have a general reduction of nerve transmission compared to control, but the facilitation of sensory evoked signaling is maintained. 

Dark-adapted ERG showed waveform changes induced by sevoflurane anesthesia in both control and diabetic groups. Profound anesthesia increased the latency and decreased the amplitude of the ERG. Our results were consistent with similar findings that indicated a decrease in the amplitude of the scotopic threshold response and increased peak latencies [**11**].

Other studies have demonstrated changes in ERG produced by volatile anesthetics especially through the delay of the b wave latency and less through amplitude decrease [**12**]. These abnormalities of the visual pathway have been shown to be persistent even 2 h after sevoflurane anesthesia [**13**,**14**].

Diabetes produced a significant delay of a and b wave latency of the ERG at 10 weeks post-STZ diabetic induction, with the exception of highly profound anesthetic depth (8% sevoflurane), where the result for the b wave are conflicting. Similar retinal dysfunctions measured by ERG alterations, that imply waveform delays, were found at 1-month post-STZ [**15**] or more [**16**]. In cases of excessive STZ administration (100 mg/ kg for 2 days), a-wave, b-wave and oscillatory potentials amplitudes were found decreased as early as 2 weeks after diabetic induction [**17**]. Other articles that showed the same finding sustain the more consistent delay of a wave produced by diabetes found in our results and no changes in b wave timing at 12 weeks of diabetes [**18**]. We found a reduction in the amplitude of the a-b wave only when ERG was recorded under 6% and 8% sevoflurane concentration. This showed a sensitivity of the ERG in diabetic animals to profound anesthesia. Some studies have found early retinal damage at only 2 weeks after diabetic induction showed by reduced amplitude of the ERG compared to control, b-wave being more affected [**19**]. Other reports that indicated opposite results found no statistical differences in a and b waves of the ERG between control and diabetic. These results were recorded in female rats, so gender should also be taken into consideration when evaluating diabetic induced abnormalities [**20**].

In conclusion, neurophysiological studies like VEP and ERG are useful in the assessment of retinal and optic nerve dysfunctions produced by diabetes, yet considering the alterations that occur during anesthesia if this is used [**3**,**21**].
